# Changes in GC-MS metabolite profile, antioxidant capacity and anthocyanins content during fermentation of fine-flavor cacao beans from Ecuador

**DOI:** 10.1371/journal.pone.0298909

**Published:** 2024-03-01

**Authors:** Ivan Chóez-Guaranda, María Maridueña-Zavala, Adela Quevedo, María Quijano-Avilés, Patricia Manzano, Juan M. Cevallos-Cevallos

**Affiliations:** 1 Escuela Superior Politécnica del Litoral, ESPOL, Centro de Investigaciones Biotecnológicas del Ecuador (CIBE), ESPOL Polytechnic University, Guayaquil, Ecuador; 2 Escuela Superior Politécnica del Litoral, ESPOL, Facultad de Ciencias de la Vida (FCV), ESPOL Polytechnic University, Guayaquil, Ecuador; University of the Philippines Diliman, PHILIPPINES

## Abstract

The fermentation of fine-flavor cacao beans is a key process contributing to the enhancement of organoleptic attributes and monetary benefits for cacao farmers. This work aimed to describe the dynamics of the gas chromatography-mass spectrometry (GC-MS) metabolite profile as well as the antioxidant capacity and anthocyanin contents during fermentation of fine-flavor cacao beans. Samples of Nacional x Trinitario cacao beans were obtained after 0, 24, 48, 72, 96, and 120 hours of spontaneous fermentation. Total phenolic content (TPC), ferric reducing antioxidant power (FRAP), and total anthocyanin content were measured by ultraviolet-visible (UV-Vis) spectrophotometry. Volatiles were adsorbed by headspace solid phase microextraction (HS-SPME) while other metabolites were assessed by an extraction-derivatization method followed by gas chromatography-mass spectrometry (GC-MS) detection and identification. Thirty-two aroma-active compounds were identified in the samples, including 17 fruity, and 9 floral-like volatiles as well as metabolites with caramel, chocolate, ethereal, nutty, sweet, and woody notes. Principal components analysis and Heatmap-cluster analysis of volatile metabolites grouped samples according to the fermentation time. Additionally, the total anthocyanin content declined during fermentation, and FRAP-TPC values showed a partial correlation. These results highlight the importance of fermentation for the improvement of the fine-flavor characteristics of cacao beans.

## Introduction

Ecuador is one of the largest producers of fine-flavor cacao (*Theobroma cacao*) worldwide [1] and around 75% of the total cacao bean exports from Ecuador have been recognized as fine-flavor [2]. The fine-flavor cacao cluster is mainly conformed by Trinitario and Criollo cacao groups, which are widely propagated in the tropical regions of Latin America [3, 4]. Nonetheless, the Nacional group which mostly grows in Ecuador [5] is also considered a fine-flavor cacao because of its distinctive aromas characterized by floral, green and spicy notes [4, 5]. Nowadays, hybrid cacao beans among Nacional and Trinitario varieties are commonly grown in Ecuador and have also shown a rich aroma profile [6].

The commercialization of fine-flavor cacao usually receives a premium over the price set by the London and New York cocoa futures markets and the final price of the cacao will largely depend on the demand for a particular quality and flavor profile [2]. However, the contribution of the metabolites produced during fermentation to the overall flavor profile of Nacional x Trinitario cacao is not fully understood. The fine-flavor attributes of cacao beans, essentially depend on the type of cacao as well as the applied fermentation, drying, and roasting techniques. Among all the cacao processing steps, fermentation and drying have shown a greater influence on the final quality of the beans than the cacao origin [7]. Similarly, previous findings revealed that precursor aroma composition of Criollo, and Trinitario beans from different origins were mostly influenced by fermentation as opposed to cacao origin and variety used [8]. During fermentation, flavor compounds that contribute significantly to the final quality of cacao derivatives are produced [9–11].

Characterizing the changes in the metabolite profile occurring during cacao fermentation have been proposed as a key strategy to understand the formation of aroma compounds [3]. In Colombia, 25 compounds were identified as possible markers of the fruity, nutty, and floral attributes occurring after 120 hours of controlled fermentation using Trinitario cacao beans [12]. Similarly, a recent study identified 20 volatiles and 48 discriminating metabolites as potential quality biomarkers and proposed a fermentation period of 96 hours for an optimum production of fine-flavor cacao [9]. The dynamics of volatiles during spontaneous fermentation of Amazonian Trinitario cacao beans revealed the development of fruity flavor precursors such as esters and a significant increase in the concentration of floral-aroma- compounds including alcohols, aldehydes, and ketones, as well as the continuous presence of floral terpenes after 66 hours of fermentation [13]. A similar study carried out using Nacional and Criollo cacao revealed the formation of fruity, green, woody, and floral aroma compounds including alcohols, hydrocarbons, and ketones after 72 hours of spontaneous fermentation [3]. Recent studies carried out using Nacional x Trinitario cacao beans showed that several yeast, lactic acid bacteria (LAB), acetic acid bacteria (AAB), and *Bacillus* spp. were capable to produce volatile compounds with floral and fruity attributes during fermentation [6]. In Ecuador, cocoa liquor and chocolate made from Nacional cacao beans have yielded a total of 55 volatile compounds including acids, alcohols, aldehydes, esters, ketones, pyrazines, furans, furanones, lactones, pyrans, pyrroles, and terpenes [5]. However, metabolite changes occurring during fermentation of Ecuadorian Nacional x Trinitario cacao beans were not reported.

Cacao beans are also a source of polyphenols with potential health benefits for consumers, but the contents of polyphenols, and their antioxidant activity has been shown to decrease after fermentation, drying, and roasting of Nacional, Criollo and Trinitario cacao [14–18]. However, the changes of metabolites, antioxidant activity and anthocyanins during fermentation of Ecuadorian Nacional x Trinitario cacao is still not clear.

The objective of this work was to study the changes on the GC-MS metabolite profile, antioxidant capacity and total anthocyanins content occurring during fermentation of Nacional x Trinitario cacao beans to understand the dynamics of antioxidants and aroma precursors.

## Material and methods

This research was authorized by the Department of Biodiversity of the Ecuadorean Ministry of the Environment under permit [No. MAE-DNB-2017-0266-O].

### Reagents and chemicals

N-Methyl-N-(trimethylsilyl)trifluoroacetamide (MSTFA), Folin-Ciocalteu´s phenol reagent, 2,4,6-Tris (2-pyridyl)-s-triazine (TPTZ), 6-hydroxy-2,5,7,8 tetramethylchroman-2-carboxylic acid (Trolox), gallic acid, ferric chloride, and methanol were from Sigma-Aldrich (St. Louis, USA). Saturated alkanes standard (C_7_-C_40_) was obtained from Supelco (Bellefonte, USA). Sodium acetate, potassium chloride, sodium hydroxide, hydrochloric acid, chloroform, and ethanol were acquired from J.T. Baker (New Jersey, USA). Hexane, sodium carbonate, and acetic acid were obtained from Fisher Scientific (Hampton, USA). A Milli-Q water purification equipment Millipore (Bedford, USA) was used to obtained ultrapure water.

### Cacao samples and fermentation process

For analysis of volatile compounds, ripe cacao pods of Nacional x Trinitario were collected in June 2016 from three cacao plantations located in Milagro, Guayas, Ecuador. All plantations were located in areas with similar soil and climate. Cacao pods were opened using a sterile knife and the beans were extracted. Then, about 5 kg of cacao beans were fermented spontaneously in a greenhouse at ambient temperature (about 35°C) for 5 days using plastic containers with draining holes as reported elsewhere [3]. Samples of fermenting beans (about 50 g) were removed at 24-hours intervals and stored at -80°C until analyzed. In total, 4 different fermentation batches were run in parallel as biological replicates.

For GC-MS liquid extracts, antioxidant capacity and anthocyanins analysis, about 500 g samples of on-farm fermented cacao beans were obtained from fermentation farms located in Milagro, Guayas, Ecuador. Spontaneous fermentation was carried out in the farms during 5 days using wooden boxes (1m^3^) arranged on 6 stair steps in which about 1000 kg of fresh cacao beans were covered with polypropylene bags. Samples were collected every 24 hours using sterile gloves, refrigerated and transported to the laboratory, stored at -80°C, and then used for analysis. A total of 3 boxes were sampled daily from each fermentation time as biological replicates [6].

### Analysis of volatile compounds

Volatile compounds were detected by headspace solid phase microextraction (HS-SPME) using the procedure reported in previous research [3]. Briefly, 5 g (about two beans) of fermented cacao beans were ground under liquid nitrogen and the powder was then transferred into a 40 mL SPME vial (27.5 x 95 mm), and incubated in a water bath for 30 minutes at 50μC. Then, a 50/30 μm Divinylbenzene/Carboxen/Polydimethylsiloxane (DVB/CAR/PDMS) bonded SPME fiber of 1 cm Supelco (Bellefonte, USA) retained in a 24 Ga manual holder was exposed to the headspace of each sample for 30 minutes at 50°C. Subsequently, the SPME fiber was removed from the vial, and injected into an Agilent Technologies (Santa Clara, USA) GC-MS system (7890A GC coupled to a 5975C MSD with triple axis detector). A capillary column DB-5MS (30 m × 0.25 mm) with phenyl dimethylpolysiloxane was used as stationary phase (0.25-micron film thickness), and ultra-high purity grade helium as the carrier gas (0.80 mL / min). The desorption of volatile compounds was done at 240°C using splitless mode, the initial oven temperature was 70°C, and the temperature programing was 70–310°C at 7°C/min. The MSD transfer line was 280°C, and the ion source temperature was 230°C. Electron ionization of 70 eV was used, and the data of compounds was collected using a frequency of 4 s−1 with the full scan mode (40–750 amu) using no solvent delay in the quadrupole mass analyzer.

### Analysis of liquid extracts

Metabolite extraction and derivatization were performed as reported elsewhere [19]. On-farm fermented cacao beans were ground under liquid nitrogen using a mortar and pestle. About 600 mg of the powder were then mixed with 1.5 mL of a methanol/ chloroform/ water (8:1:1 v/v) solution followed by maceration for 48 hours at 7°C. Then, extracts were centrifuged for 4 minutes at 15000 rpm in a Heraeus Multifuge X1R Thermo Scientific (Waltham, USA), and the supernatant was taken to a water bath VWR Scientific Products (Cornelius, USA) at 80°C to dryness. Dried extracts were then mixed with 150 μL of MSTFA, and incubated in a water bath for 90 minutes at 80°C. Then, 1 μL of the suspension was injected into the GC-MS. The injection was carried out at 250°C using splitless mode, and ultra-high purity grade helium as the carrier gas (1 mL/min). The oven temperature was started at 80°C for 1 minute, then it was increased to 300°C at 7°C/min, and was maintained to 300°C for 5 minutes. Other GC-MS parameters were carried out exactly as explained in the volatiles section.

### Data processing, compounds identification and aroma descriptors

GC-MS data analysis was done as described in previous studies [3]. In general, GC-MS runs showed that peaks were too close to each other throughout the chromatogram, so no internal standard was used to prevent overlapping or interference with the metabolites from the sample. Additionally, the use of internal standards may not be adequate for the normalization of metabolites belonging to different chemical classes, as intensity level fluctuations may differ between various classes [20]. Therefore, median fold change normalization was carried out in all samples as the suggested workflow when internal standard normalization is not recommended. Briefly, three metabolites with uniform intensities across all samples were selected and the fold change of each metabolite was calculated using one of the samples as the reference. The medians of the fold changes were calculated for each sample and used as the normalization factor for the sample [20–22].

Also, quality control (QC) measures as well as reproducibility assessment of SPME and liquid extracts were executed by running one selected sample after 5 consecutive sample runs and estimating the variations in retention time and peak areas. Maximum acceptable coefficient of variation was 30% for a given metabolite in QC runs [19, 23].

Raw data files were obtained in NetCDF/AIA (*.cdf) format using ChemStation software E.02.02 (Agilent Technologies). After that, MzMine 2 software was used for raw data processing including baseline correction, noise filtering, peak detection, chromatographic building, spectral deconvolution, and alignment based on peak areas as well as retention times [24]. Then, Log 2 fold change (FC) values were used to express metabolites variations of five-days fermented vs. unfermented cacao after data normalization [25]. In addition to deconvolution process, the mass spectra were compared at the beginning and the end of each chromatographic peak to confirm peak purity as a means to detect metabolite co-elution [26]. No metabolite coelution was detected and compounds were putatively identified by matching mass spectra with the National Institute of Standards and Technology (NIST) 11 and Wiley 9 databases. Database compounds showing forward and reverse matching scores above 700 were selected and their identity was confirmed by comparing the linear retention index of each metabolite with that of the pure standards using a series of saturated *n*-alkanes (C_7_-C_40_) and a tolerance of 10 index units. Finally, The Good Scents Company database (http://www.thegoodscentscompany.com) [27] was used to obtain aroma descriptors of volatile compounds. The Good Scents Company is considered on par with other established sources of aroma data such as Flavornet and NIST, and is recognized as a reliable resource in the field [28–34].

### Total phenolic content (TPC) assay

On-farm fermented cacao beans were dried in a convection oven for 52 hours at 60°C, peeled, pulverized, and defatted using hexane. Extraction was carried out by mixing 100 mg of defatted cacao beans with 1.5 mL of a methanol/water/acetic acid solution (79.2: 20: 0.8 v/v) followed by sonication for 60 minutes using an ultrasonic bath. Extracts were then centrifuged for 10 minutes at 4400 rpm, the supernatants were obtained, and kept in the dark at -20°C until analyzed. The TPC was determined using the Folin-Ciocalteu method as reported elsewhere [35–37]. Briefly, 20 μL of the standard or extract (supernatant) was mixed with 1580 μL of water, 100 μL of Folin-Ciocalteu reagent, and 300 μL of 20% sodium carbonate and incubated for 2 hours in the dark prior to measuring the mixture’s abosorbance at 765 nm. Gallic acid (50–500 μg/mL) was used as the standard, and the results were expressed as milligrams of Gallic Acid Equivalents (GAE) per gram of defatted cacao bean (DCB).

### Ferric reducing antioxidant power (FRAP) assay

The FRAP was determined by assessing the sample’s ability to reduce the ferric-tripyridyl-triazine (Fe3+-TPTZ) complex to the ferrous form (Fe2+) as reported elsewhere [38, 39]. Briefly, the (Fe^3+^-TPTZ) complex was prepared by mixing 300 mM acetate buffer (pH 3.6), 10 mM TPTZ solution prepared in 40 mM hydrochloric acid, and 20 mM ferric chloride (10:1:1 v/v). Then, 10 μL of the standard or extract (supernatant) was mixed with 300 μL of the (Fe^3+^-TPTZ) complex. The mixture was then incubated at 37°C for 30 minutes and the absorbance was measured at 593 nm. Trolox (25–250 μg/mL) was used as the standard, and the results were expressed as mg of Trolox equivalents (TE) per gram of defatted cacao bean (DCB). TPC and FRAP assays were conducted in a Biotek Synergy HTX multi-mode microplate reader with UV-Vis detector (Vermont, USA).

### Total anthocyanin content assay

The total anthocyanin content was determined by the pH-differential procedure [40]. First, the defatted cacao beans were suspended in both, hydrochloric acid potassium chloride (0.025 M, pH 1.0), and sodium acetate (0.4 M, pH 4.5) buffers. The suspensions were then filtered, centrifuged and the absorbance was measured at both 520, and 700 nm using a Thermo Spectronic Genesys 5 spectrophotometer (Waltham, USA). Then, the total anthocyanin content was calculated using the Eq 1:

$$Total\;anthocyanin\;content\;(mg/g)=\frac{A\;x\;MW\;x\;DF\;x\;V\;x\;10^{3}}{ɛ\;x\;l\;x\;M}$$ (1)

where, A = (Absorbance_520nm_−Absorbance_700nm_)pH_1.0_ − (Absorbance_520nm_ − Absorbance_700nm_) pH_4.5_, MW = molecular weight of cyanidin-3-glucoside (449.2 g/mol), DF = dilution factor, V = volume of the aliquot, ɛ = molar extinction coefficient of cyanidin-3-glucoside (29,600), l = path length (1 cm), and M = weight of sample (g). Finally, the results were expressed as mg cyanidin-3-glucoside Equivalents (CGE) per gram of defatted cacao bean (DCB). All colorimetric experiments were performed by triplicate, and results were expressed as mean ± standard error.

### Statistical analysis

Principal components analysis (PCA) and heatmap cluster analysis (HCA) were performed to compare the overall GC-MS metabolite profile of the samples during the entire fermentative process. Additionally, repeated measures analysis was carried out to assess the effect of the fermentation time on the levels of each metabolite. Compounds showing a significant (p < 0.05) time effect after the repeated measures analysis were regarded as significant metabolites.

Analysis of variance (ANOVA) and Tukey test were performed for comparison of response values among fermentation days with 5% of significance as well as Pearson´s correlation coefficient was used to measure relationship between values of the spectrophotometric assays. All data were processed in XLSTAT 2020 (Addinsoft) software package for Microsoft Excel. Furthermore, the resulting data were imported to Heatmapper to perform heatmap analysis [41].

## Results and discussion

Cacao fermentation is a key step of the chocolate production process, and a series of reactions occur to improve the sensory quality of cacao beans. In this study the dynamics of volatile and several non-volatile compounds occurring during fermentation of Nacional x Trinitario is presented. Non-volatile compounds in the liquid extracts were assessed under normal fermentation settings, but volatile compounds were investigated using small-scale fermentations in plastic containers to prevent the loss of volatile compounds that may occur in samples from remote fermentation areas. Although cacao fermentation has been reported in vessels of different sizes and materials, further research is needed to assess the effect of cacao mass and type of vessel on the dynamics of cacao fermentation.

### Analysis of volatiles

During the cacao bean fermentation, a total of 57 volatile organic compounds were identified, including 9 volatile acids, 12 alcohols, 14 aldehydes and ketones, 2 amines, 4 esters, 1 furan, 7 hydrocarbons, 1 sulfur compound, and 7 other compounds. The Log2 (FC) values corresponding to the comparison of fermented samples vs. unfermented samples, and the aroma descriptors related to the detected compounds are described in Table 1 in alphabetical order. Similarly, the data processing, compounds identification and aroma descriptors of the metabolites detected in the headspace of cacao samples at different fermentation times is detailed in S1 Table. These findings are similar to a previous report describing 62 volatile compounds in Nacional and Criollo cacao beans from the same origin [3]. Differences between the aforementioned cacao varieties have been outlined by the presence of 1-phenylpent-4-en-2-ol, 2-octenyl-acetate, germacrene, and hexanal in Criollo beans [3]. These four metabolites were also not detected in the Nacional x Trinitario samples of the present study.

**Table 1:** Aroma descriptors and volatiles compounds detected in the headspace of cacao samples at different fermentation times.

Compound	Log2 (FC)^a^					Linear retention index	Aroma Descriptor
	Fermentation time (days)						
	1	2	3	4	5		
1-amino-1-ortho-chlorophenyl-2-(2-quinoxaliny)ethene	0.08	-0.12	0.02	0.01	0.42	1008	NF
1-Butanol	NDZ	NDZ	NDZ	NDZ	NDZ	801	Fruity, wine
1-Octanol, 2-butyl-	NDZ	NDZ	NDZ	NDZ	NDZ	1312	NF
1,3-Bis-t-butylperoxy-phthalan	NDFT	NDFT	NDFT	NDFT	NDFT	1191	NF
2-Acetoxytetradecane	-0.71	-0.15	-1.77	-1.47	0.44	1248	NF
2-Butanone	NDZ	NDZ	NDZ	NDZ	NDZ	737	Ethereal, slightly fruity
2-Heptanol	-0.36	-0.53	NDFT	-0.82	-2.71	918	Citrus, fruity
2-Heptanol, acetate	-1.25	-0.50	-0.25	-1.93	-1.45	1054	Fenugreek fruity
2-Heptanone	1.96	4.30	3.40	3.00	3.85	914	Cheese, fruity
2-Methyl-5H-dibenz[b,f]-azepine	NDZ	NDZ	NDZ	NDZ	NDZ	859	NF
2-Nonanone	0.52	2.58	2.40	1.58	1.74	1109	Fruity
2-Octanol, acetate	-18.48	NDFT	3.21	-2.22	NDFT	1152	Fruity
2-Pentanol	NDZ	NDZ	NDZ	NDZ	NDZ	705	Green, banana, fermented, fruity
2-Pentanol, acetate	-1.75	NDFT	NDFT	0.69	NDFT	875	Fruity, herbal
2-Pentanone	NDZ	NDZ	NDZ	NDZ	NDZ	689	Fruity
2-Pentanone, 5-phenyl-	NDZ	NDZ	NDZ	NDZ	NDZ	1261	Floral, floral sweet pea
2-Undecanone	NDZ	NDZ	NDZ	NDZ	NDZ	1307	Fruity
2,3-Butanediol	2.20	3.77	2.77	0.96	2.72	821	Fruity, creamy, buttery
2,4-Diacetoxypentane	2.93	NDFT	NDFT	NDFT	NDFT	1131	NF
3-Formyl-N-methyl-9-[phenylethynyl]dibenzo[2,3-a:5,6-a](1,4)-thiazine	0.43	-1.34	0.64	NDFT	NDFT	1261	NF
3-Octen-1-ol	NDZ	NDZ	NDZ	NDZ	NDZ	1291	Fruity
3-Trifluoroacetoxypentadecane	NDZ	NDZ	NDZ	NDZ	NDZ	1674	NF
3,6-Heptanedione	-1.70	-1.69	-1.94	-1.66	-1.69	1040	NF
4-Hexanoic acid	NDFT	NDFT	NDFT	NDFT	NDFT	1574	Fatty
5-Hepten-2-ol	-1.70	NDFT	NDFT	NDFT	NDFT	838	NF
6-Undecylamine	NDZ	NDZ	NDZ	NDZ	NDZ	1894	NF
Acetic acid	NDZ	NDZ	NDZ	NDZ	NDZ	527	Pungent, sour, vinegar
Aromadendrene	NDZ	NDZ	NDZ	NDZ	NDZ	1520	Woody
Benzaldehyde	0.10	-2.52	-2.59	-1.36	-0.56	987	Almond, cherry, fruity
Benzeneacetaldehyde	1.08	-0.51	-1.06	0.20	0.39	1066	Floral, green
Benzeneethanol	1.66	1.35	2.26	2.89	2.99	1135	Floral, rose
Benzoic acid, pentyl ester	-0.19	4.02	NDFT	-3.21	-0.23	1411	Floral, green
Benzyl hydrazine	NDZ	NDZ	NDZ	NDZ	NDZ	1147	NF
Butanal	-1.94	-2.67	-2.55	-2.57	-3.04	632	Pungent cacao, chocolate
Butanoic acid	NDZ	NDZ	NDZ	NDZ	NDZ	861	Cheese, buttery, fruity
Caffeine	NDZ	NDZ	NDZ	NDZ	NDZ	1871	Odorless
Carbamic acid	NDZ	NDZ	NDZ	NDZ	NDZ	423	NF
Cyclohexanol	1.05	0.36	-0.67	-0.30	0.14	839	Camphoraceous
Decanal	NDZ	NDZ	NDZ	NDZ	NDZ	1220	Orange peel, citrus, floral
Epoxylinalool	NDZ	NDZ	NDZ	NDZ	NDZ	1191	Floral, honey
Ethanol	-3.52	NDFT	NDFT	NDFT	NDFT	433	Alcoholic
Ethanone, 1-phenyl	0.13	-0.17	-0.01	-0.34	0.07	1102	Almond, floral
Heptanoic acid	NDZ	NDZ	NDZ	NDZ	NDZ	902	Cheesy
Hexanoic acid	NDZ	NDZ	NDZ	NDZ	NDZ	953	Fatty
n-Hexylpentanamide	NDFT	NDFT	NDFT	NDFT	NDFT	1610	NF
L-Linalool	0.24	0.47	1.68	0.38	0.88	1118	Floral, lavender
Methane, thiobis-	NDFT	NDFT	NDFT	NDFT	NDFT	506	Sulfurous
Naphthalene	0.47	-0.67	0.37	-1.26	-1.70	1209	Dry, pungent, tarry
Pentanal	NDZ	NDZ	NDZ	NDZ	NDZ	830	Fermented, fruity, nutty
Pentanoic acid	NDZ	NDZ	NDZ	NDZ	NDZ	942	Cheesy, Sweaty
Peracetic acid	NDZ	NDZ	NDZ	NDZ	NDZ	574	Pungent, stinging sour
Propanoic acid	NDZ	NDZ	NDZ	NDZ	NDZ	807	Pungent acidic and dairy-like
Tetramethoxyisoquino(1,2-b)quinazolin-8-one	1.57	0.71	1.29	0.38	0.28	1990	NF
Trimethylacenaphthylene	NDZ	NDZ	NDZ	NDZ	NDZ	1475	NF
Trimethyltetrahydrofuran	NDZ	NDZ	NDZ	NDZ	NDZ	846	NF
Valencene	NDZ	NDZ	NDZ	NDZ	NDZ	1348	Citrus, fruity
α-Copaene	0.34	2.89	NDFT	0.89	2.02	1398	Spicy, woody

^a^Fold change (FC) obtained by dividing the intensity of each metabolite from fermented samples (1 to 5 days) by that of unfermented samples and expressed as Log2 (FC).

NDZ = Metabolite not detected in unfermented samples. No Log2 (FC) was calculated.

NDFT = Metabolite not detected at the fermentation time. No Log2 (FC) was calculated

NF = Aroma descriptor not found

A recent study identified 55 volatiles in Nacional beans from the Amazon region processed into cocoa liquor [5]. However, only 5 alcohols (L-linalool, ethanol, 2,3-butanediol, 2-pentanol, 2-heptanol), 3 ketones (2-butanone, 2-heptanone, 2-nonanone), 1 aldehyde (benzaldehyde) and acetic acid were also detected in the current research using fermented Nacional x Trinitario beans. Results suggest that flavor compound development is also influenced by the drying, roasting and alkalization processes [4] that are commonly applied after fermentation and during the production of cocoa liquor. Additionally, volatile profile variations could be associated to the geographical origin of the beans [4], and the microbial community present under each environmental condition [42].

**Fig 1:**
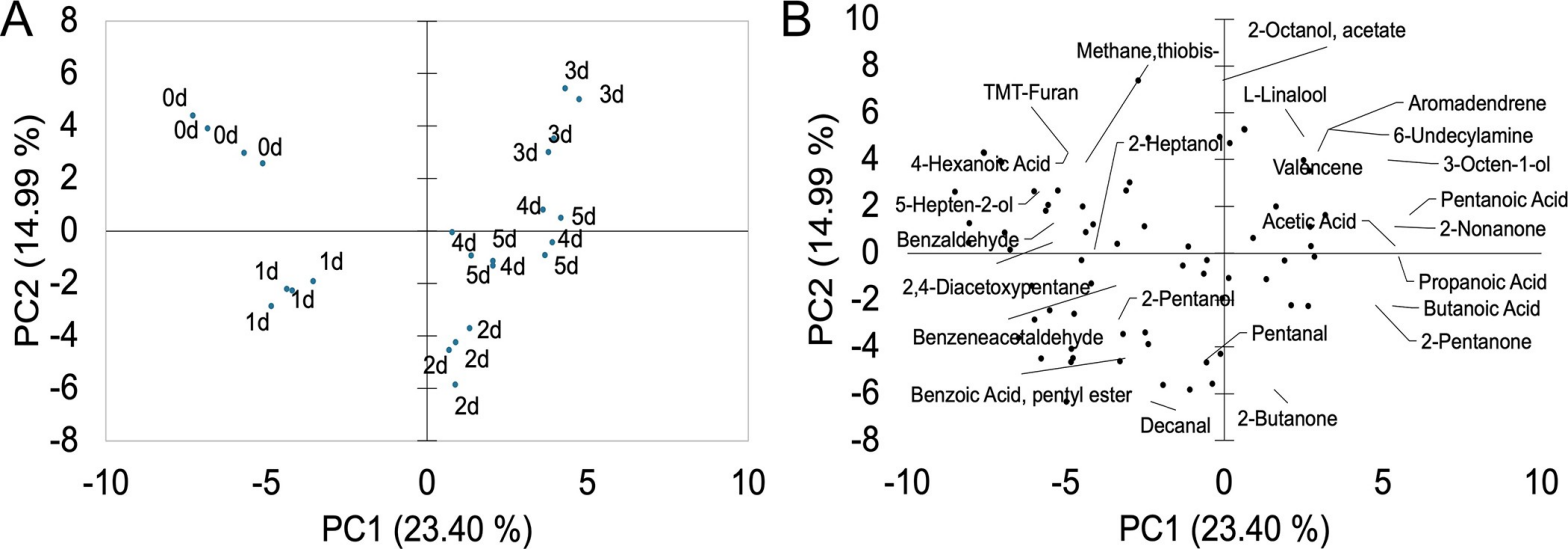
Principal components analysis (PCA) of volatile compounds. Score plot (A) shows all samples and Loading plot (B) shows the metabolites with the highest loadings. Values in parenthesis represent the percentage contribution of PC1 and PC2 to the total variance.

**Fig 2:**
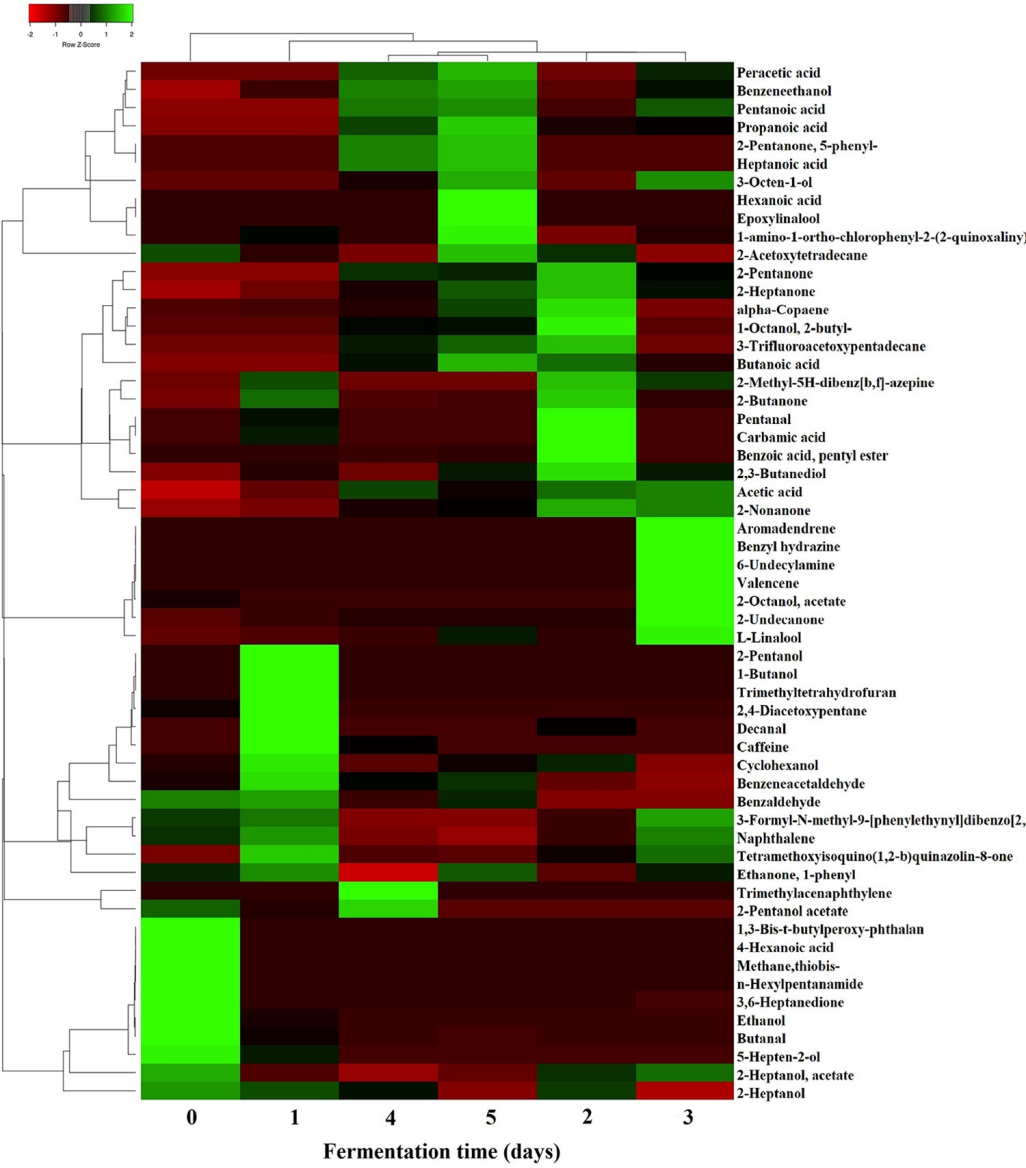
Heatmap analysis of volatile compounds during fermentation. The Heatmap illustrates the changes in the content of identified volatiles through 5-days of cacao bean fermentation.

Principal components analysis (PCA) showed a marked grouping of samples according to the fermentation time. Samples fermented for 0, 1, 2 and 3 days grouped in separate clusters while samples fermented for 4 and 5 days formed one cluster (Fig 1A). Similarly, the HCA showed that samples from days 0 and 1 were separated from the rest, while samples from days 2 and 3 as well as days 4 and 5 clustered together (Fig 2). PCA attempts to reduce the dimensionality of the data while maximizing the explained variance, whereas HCA performs distance calculations among all samples. Despite the differences in the calculation methods, similar clusters were obtained by both PCA and HCA. Fig 1B shows the loading plot of the metabolites with the highest contribution to the clustering. Before the start of the fermentation, the beans were principally characterized by the presence of 3 alcohols (2-heptanol, 5-hepten-2-ol, ethanol), 2 aldehydes (benzaldehyde, butanal), one ester (2-heptanol acetate), one amine (n-hexylpentanamide), one ketone (3,6-heptanedione), one acid (4-hexenoic acid), and one sulfur (methane,thiobis-) and 1,3 Bis-t-butylperoxy-phtahlan. Butanal has been reported in unfermented Criollo and Nacional cacao and it has been related to fruity, green, and banana aromatic notes [3]. Similarly, ethanol has been described in fresh cacao beans of Criollo, Nacional and Trinitario groups and it has been associated with sweet and alcoholic odor descriptions [3, 13]. After the first day of fermentation, volatiles were mainly characterized by 3 aldehydes (benzaldehyde, benzeneacetaldehyde, decanal), 3 alcohols (1-butanol, 2-pentanol, cyclohexanol), 1 furan (trimethyltetrahydrofuran), 1 hydrocarbon (2,4-diacetoxypentane), and caffeine. Decanal has been linked to sweet aldehydic, orange peel, and citrus floral aromatic features, and has been reported in fermented cacao clones from Brazil [34]. Caffeine is one of the alkaloids responsible for the bitter taste of cacao beans [5], it is more abundant in fine-flavor than bulk cacao [4], and has previously been identified during fermentation of Criollo and Nacional cultivars [3]. During the following days of fermentation, distinctive flavor precursor constituents were produced, and samples were clustered in the heatmap according to the fermentation time (Fig 2). At the end of the second day, aroma compounds of cacao samples were mostly associated with 3 ketones (2-butanone, 2-pentanone, 2-heptanone), 1 hydrocarbons (α-copaene), 2 alcohols (2,3-butanediol, 1-octanol,2-butyl-), 1 ester (benzoic acid, pentyl ester), 1 aldehyde (pentanal), 1 acid (carbamic acid), and 2-methyl-5H-dibenz[b,f]-azepine (Fig 2). Particularly, the sesquiterpene α-copaene has been related to woody and spicy aromatic characteristics [3, 34]. After the third day of fermentation, other volatiles were formed, including 3 ketones (2-nonanone, 2-undecanone, tetramethoxyisoquino(1,2-b)quinazolin-8-one, 2 hydrocarbons (aromadendrene, valencene), 2 alcohols (L-linalool, 3-octen-1-ol), 2 esters (2-heptanol acetate, 2-octanol acetate), 1 acid (acetic acid), 1 hydrazine (Benzyl hydrazine), and 1 amine (6-undecylamine). L-Linalool has predominantly been associated with floral and green aromatic notes, and has been detected in different cacao clones [34] and varieties [3, 13]. Subsequently, volatiles compounds during the fourth and fifth day of fermentation were principally constituted by 6 acids (butanoic acid, heptanoic acid, hexanoic acid, peracetic acid, pentanoic acid, propanoic acid), 2 alcohols (benzeneethanol, epoxylinalool), 1 ester (2-pentanol acetate), 1 ketone (2-pentanone, 5-phenyl-), and 1 hydrocarbon (trimethylacenaphthylene). Epoxylinalool, and benzeneethanol have been largely associated to floral and honey aroma descriptors [3, 13, 34], while 2-pentanone, 5-phenyl- has been reported in roasted dark chocolate prepared with Forastero cacao beans [43].

In general, the dynamic of main volatiles was defined by the degradation of aldehydes, and sulfurs from the start of fermentation, the formation of organic acids, amines, esters, and ketones from second day to the following days of fermentation, as well as the persisting presence of alcohols and hydrocarbons during the entire fermentative process. The production of alcohols, esters, and ketones have been mainly attributed to yeasts [42], and derived from fatty acids, amino acids or esterification reactions [13, 44]. Ketones mainly provide fruity and floral odor attributes while alcohols contribute to desirable floral and sweet notes which is characteristic of high-quality fermented cacao beans. Meanwhile, esters have been associated with fruity aroma descriptors in fermented and roasted cacao beans [34].

### Dynamics of individual volatiles

**Fig 3:**
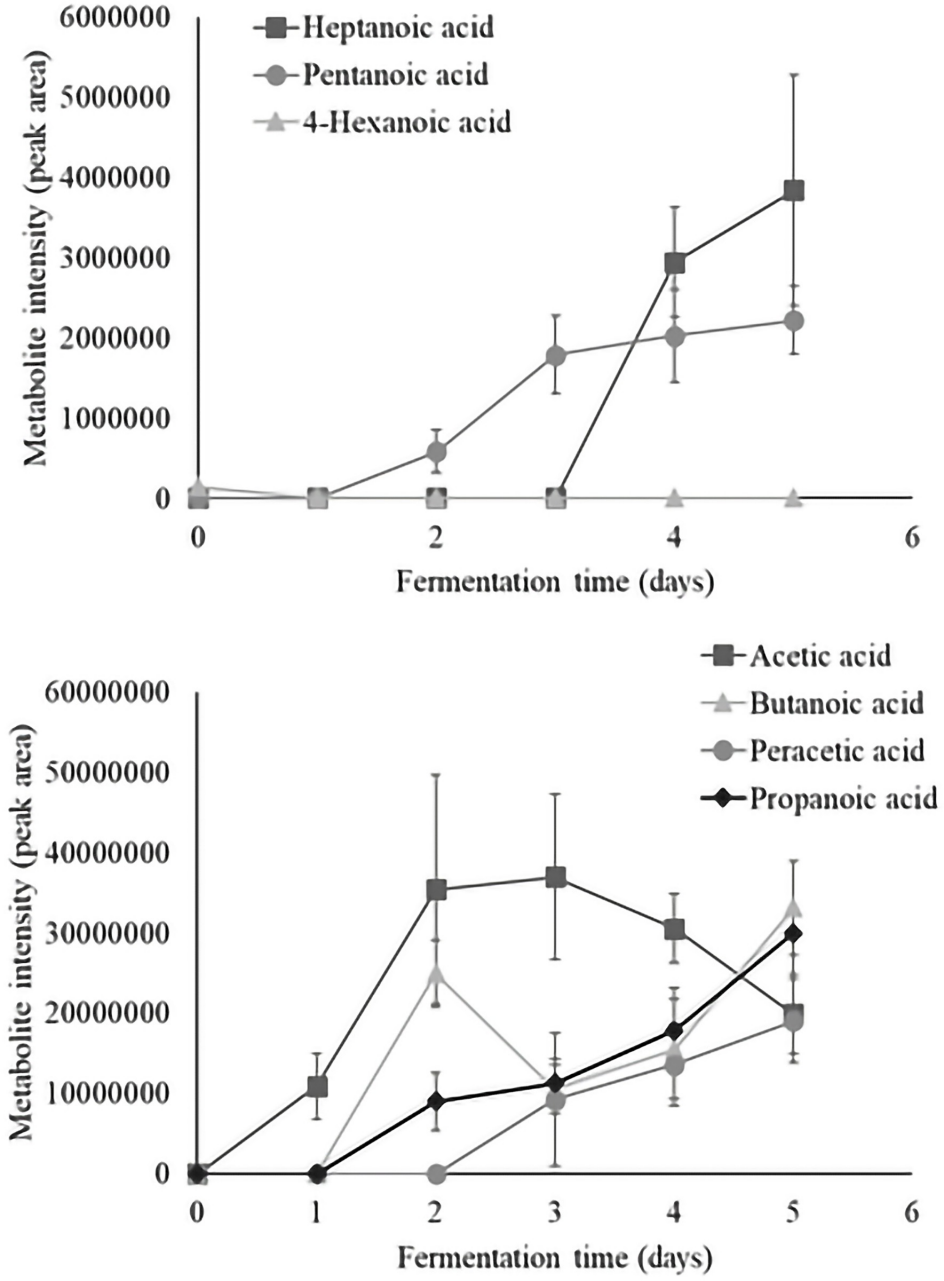
Dynamics of the organic acids which content significantly changed during fermentation (*P*<0.05). Levels of each significant organic acid are presented as metabolite intensity (peak area) through 5-days of cacao bean fermentation.

**Fig 4:**
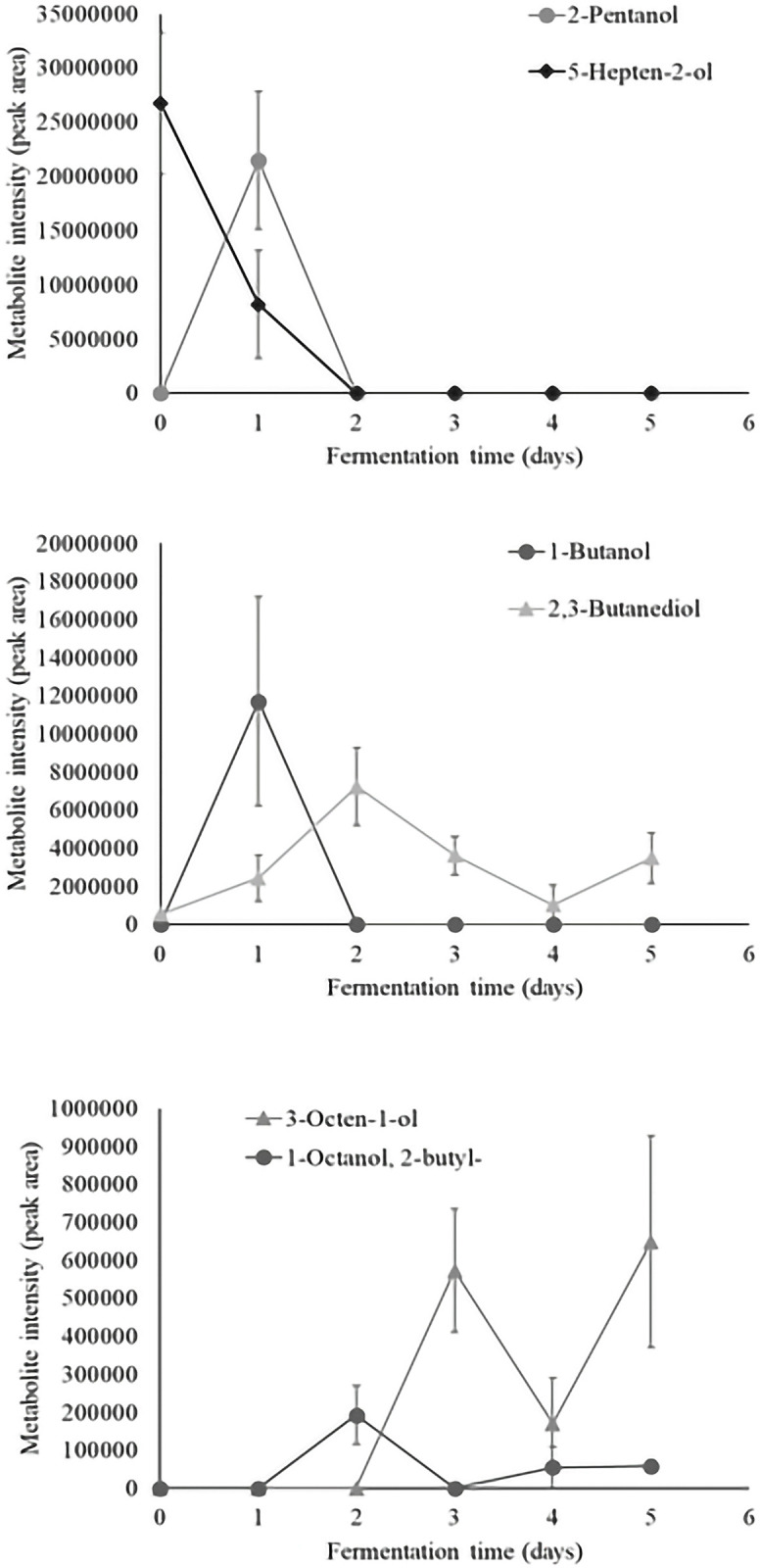
Dynamics of the alcohols which content significantly changed during fermentation (*P*<0.05). Levels of each significant alcohol are presented as metabolite intensity (peak area) through 5-days of cacao bean fermentation.

**Fig 5:**
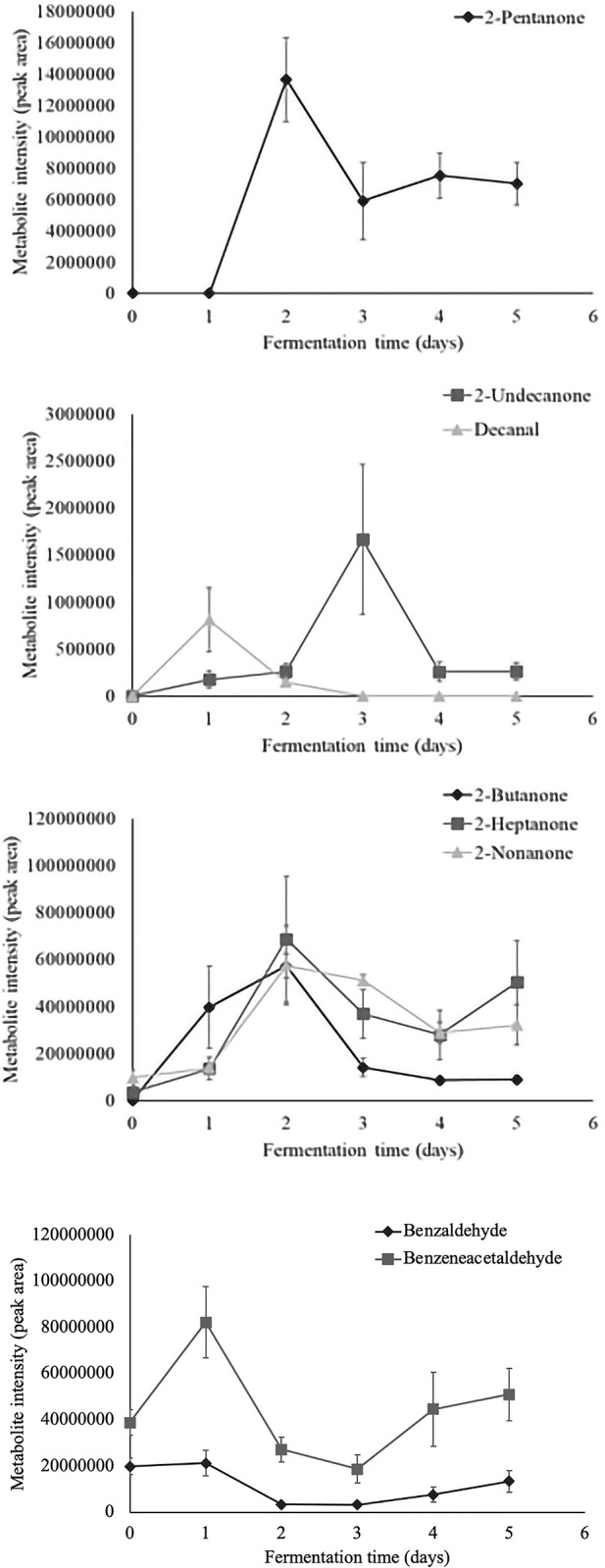
Dynamics of the aldehydes and ketones which content significantly changed during fermentation (*P*<0.05). Levels of each significant aldehyde and ketone are presented as metabolite intensity (peak area) through 5-days of cacao bean fermentation.

**Fig 6:**
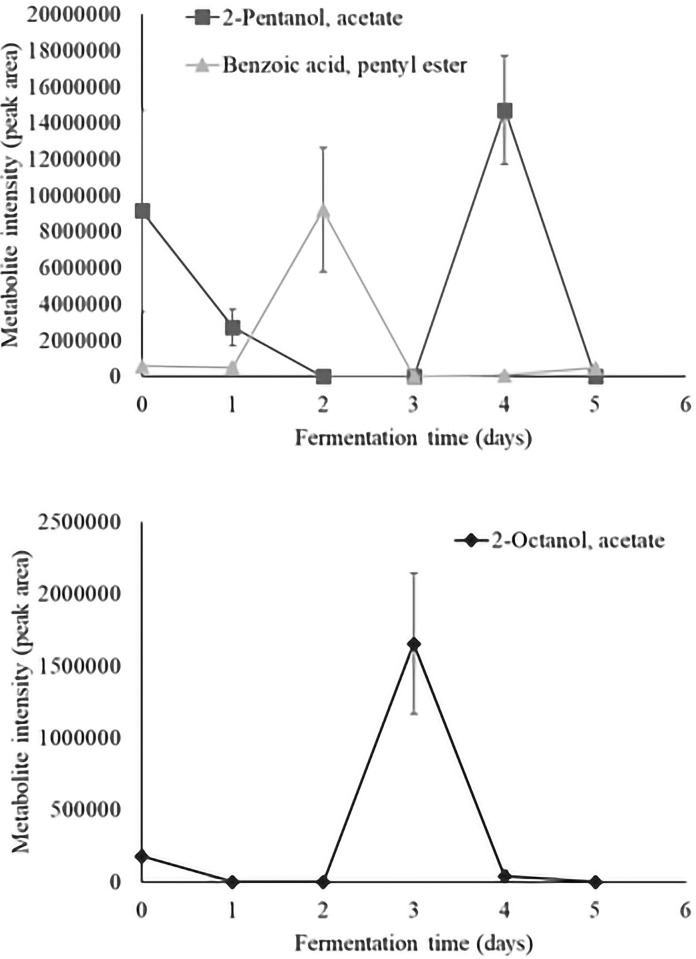
Dynamics of the esters which content significantly changed during fermentation (*P*<0.05). Levels of each significant ester are presented as metabolite intensity (peak area) through 5-days of cacao bean fermentation.

**Fig 7:**
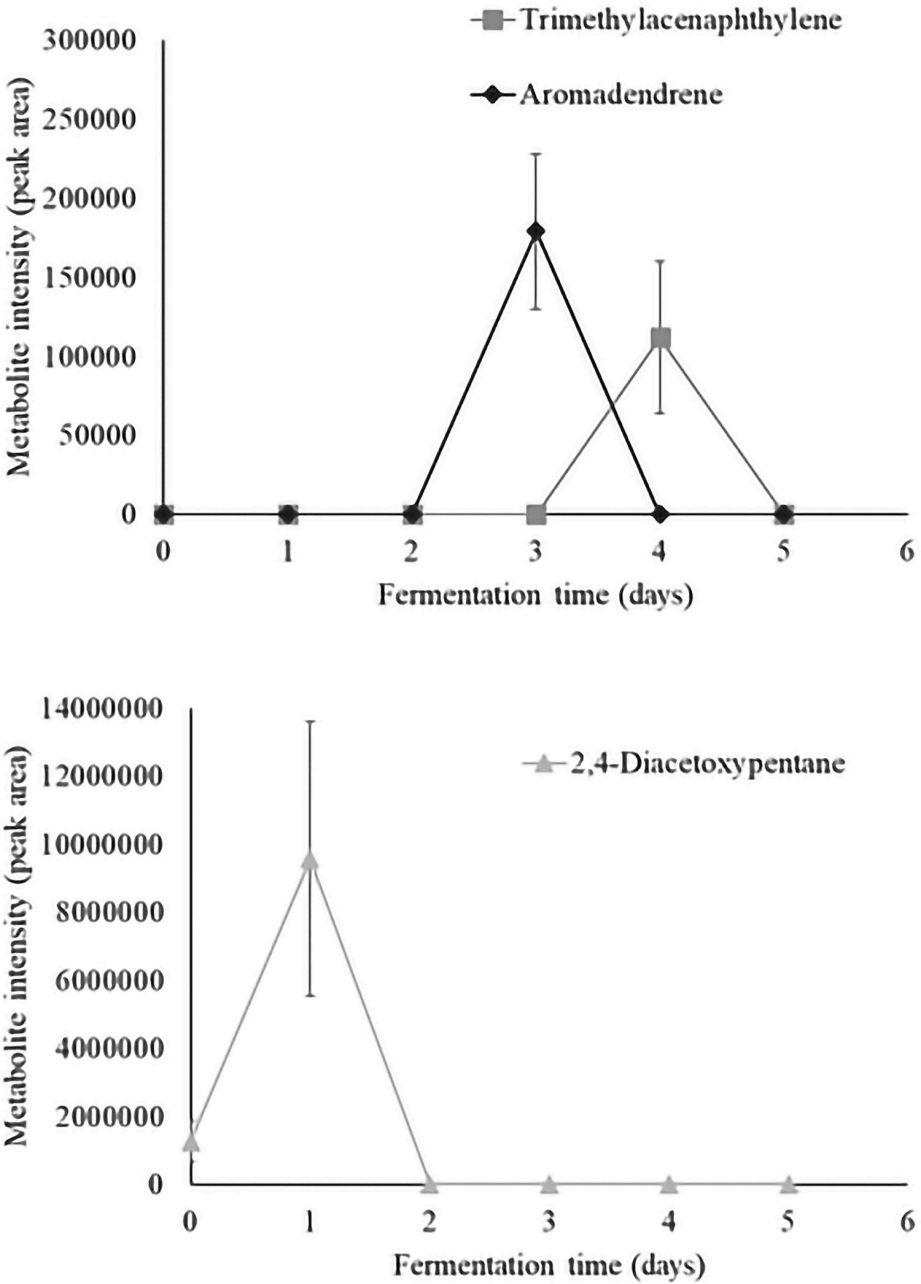
Dynamics of the hydrocarbons which content significantly changed during fermentation (*P*<0.05). Levels of each significant hydrocarbon are presented as metabolite intensity (peak area) through 5-days of cacao bean fermentation.

**Fig 8:**
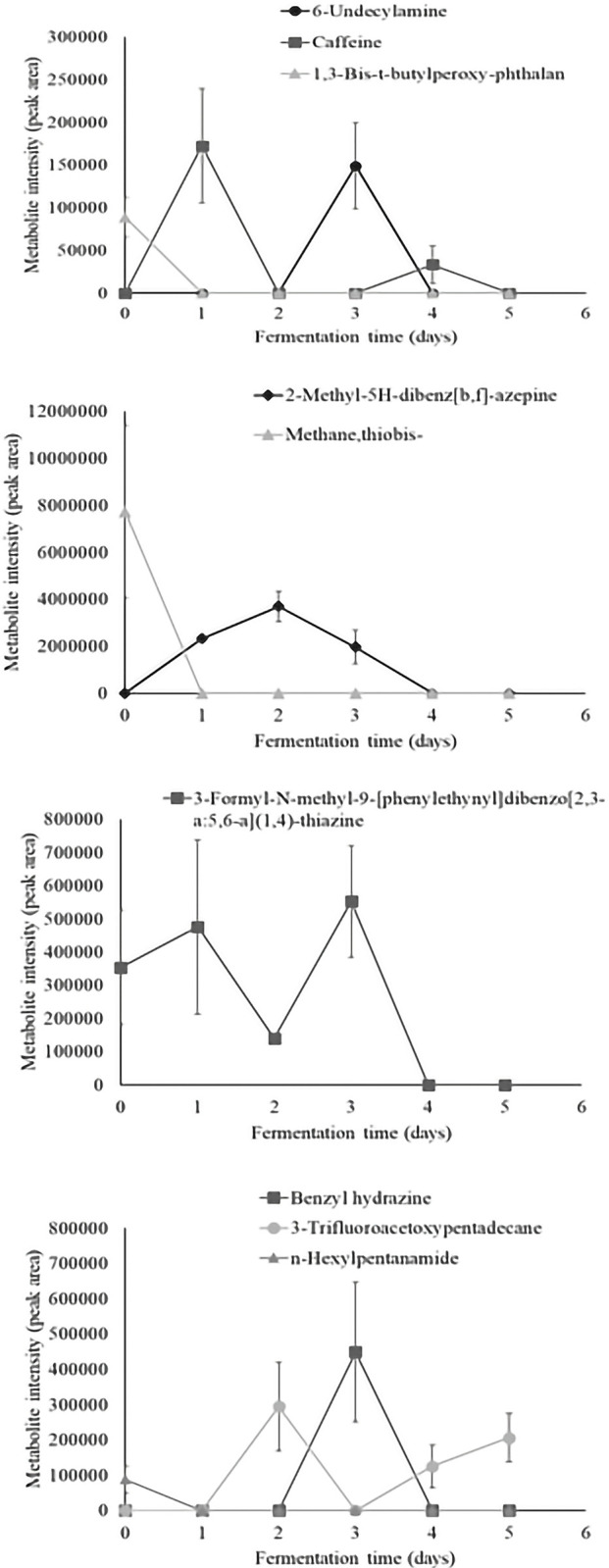
Dynamics of the other significant volatiles which content significantly changed during fermentation (P<0.05). Levels of each significant other volatile are presented as metabolite intensity (peak area) through 5-days of cacao bean fermentation.

Time series analysis of individual volatiles revealed a significant time effect in 36 aroma compounds. Metabolite intensity values of significant volatiles are illustrated as metabolite intensity (peak area) means ± standard error (n = 4) in Figs 3–8, and include 7 organic acids, 6 alcohols, 8 aldehydes and ketones, 3 esters, 3 hydrocarbons, among other 9 volatiles which dynamics are explained below.

Among the organic acids which content significantly changed during fermentation (Fig 3), only 4-hexanoic acid was detected in unfermented cacao beans, and its presence was observed up to the end of first day of fermentation. Meanwhile, acetic acid was formed within the first 24 hours of fermentation, being acetic acid one of the most abundant compound. However, acetic acid content decreased after 72 hours of fermentation. The production of acetic acid is a key indicator of cacao fermentation effectiveness, and plays an important role in the development of desirable attributes of cacao-product flavors [45]. On the contrary, butanoic acid, pentanoic acid and propanoic acid were produced after 24 hours of the fermentative process. Similarly, peracetic acid, and heptanoic acid were formed after the second, and third day of fermentation, respectively; and the abundance of these compounds increased until the end of the fermentation. The propanoic acid and acetic acid have been reported for Mexican Criollo fermented cacao beans, and they have been mainly related to fruity/pungent and sour/rancid/vinegar-like odor characteristics, respectively [46]. The formation of remaining organic acids has been earlier described for Criollo and Nacional varieties, and similar odor descriptors have been reported [3]. In fact, the production of butanoic acid in cacao beans of Nacional x Trinitario has been ascribed to the influence of *Candida metapsilosis* under aerobic conditions [6]. On the whole, the formation of volatile organic acids could be attributed to the effect of acetic acid bacteria and lactic acid bacteria on alcohols produced during yeast fermentation [46].

The dynamics of alcohols which content significantly changes during fermentation are illustrated in Fig 4. The 5-hepten-2-ol and 2,3-butanediol were found in unfermented cacao beans. The 2,3-butanediol content fluctuates during the complete process but the 5-hepten-2-ol only appears until the end of second day of fermentation. Similarly, the 1-butanol and 2-pentanol were formed through the first 24 hours of fermentative process and their content decline until the end of 48 hours of fermentation. In particular, the 1-ocatanol, 2-butyl- and 3-octen-1-ol were produced after 24 and 48 hours of fermentation, respectively and their presence as well as the 2,3-butanediol could be observed until the end of fermentation. The metabolite 2,3-butanediol has been suggested as a precursor of methyl pyrazines which are indicators of good quality fermented-dried beans [13]. The metabolite 2,3-butanediol has been identified in fermented [3, 6, 34, 46], and fermented-dried-roasted cacao beans [10], including the varieties Criollo, Nacional, Trinitario, and Nacional x Trinitario as well as cacao-products [5]. Similarly, the metabolite 3-octen-1-ol has been related to earthy, fruity, green, and oily aromatic notes, and has previously been detected in fermented fine-flavor cacao beans [3] and dark chocolate [47] from Ecuador and Peru, respectively. Additionally, the formation of alcohol compounds with sweet, fruity and buttery characteristics has been attributed to *Bacillus* and *Saccharomyces* species during fermentation [3, 11, 13, 47].

Regarding aldehydes and ketones which content significantly changed during fermentation (Fig 5), the identified compounds mostly contribute fruity and almond aromas (Table 2). The metabolites 2-heptanone, 2-nonanone, benzaldehyde, and benzeneacetaldehyde were identified in unfermented cacao beans, suggesting that these are endogenous compounds that are released from cacao seeds at the beginning of the fermentation [13]. Additionally, the increment in the content of the mentioned aldehydes after 48 hours of fermentation was probably caused by yeast such as *Candida metapsilosis* and *Saccharomyces cerevisiae*, acetic acid bacteria such as *Acetobacter pasteurianus*, and lactic acid bacteria like *Liquorilactobacillus nagelii* [6]. Additionally, benzaldehyde, and benzeneacetaldehyde have been reported as detectable molecules during the transformation from unfermented cacao beans to chocolate [9, 48].

**Table 2:** Metabolites detected in liquid extracts of cacao samples at different fermentation times.

Compound	Log2 (FC)^a^					Linear retention index
	Fermentation time (days)					
	1	2	3	4	5	
1H-Indole-3-acetic acid	2.17	1.31	3.32	NDFT	1.31	1867
2-keto-D-gluconic acid	-0.60	-2.69	-0.54	NDFT	-0.61	2151
2-keto-L-gluconic acid	0.46	-1.59	-0.02	NDFT	-0.58	2162
3-α-Mannobiose	-0.26	0.42	0.15	0.84	-0.16	2635
4-Acetyl-3’-(trifluoromethyl)-diphenylamine	NDZ	NDZ	NDZ	NDZ	NDZ	1865
9,12,15-Octadecatrienoic acid	NDZ	NDZ	NDZ	NDZ	NDZ	2827
15-Deoxy-12-hydroxy-10-(trifluoromethyl)D(7)-PGA(1) methyl ester	-0.87	-1.20	NDFT	NDFT	-0.54	2609
Acetophenone	-1.72	-0.30	-1.56	-2.20	-3.52	1869
Aconitic acid	NDZ	NDZ	NDZ	NDZ	NDZ	1742
Alanine	NDFT	1.17	NDFT	NDFT	-1.27	1934
Anhydro-D-sorbitol	-4.43	-2.85	-4.68	-2.79	-4.68	1844
Anoditol	NDFT	NDFT	-0.13	1.11	-0.62	1717
Arabinopyranose	0.63	1.56	1.30	0.66	0.94	1789
Barbituric acid	0.28	-0.77	0.34	1.47	2.31	2771
Caffeine	NDZ	NDZ	NDZ	NDZ	NDZ	1840
Citric acid	-2.01	-4.94	-1.34	-1.24	-2.45	1814
Cystathionine	NDFT	0.52	NDFT	NDFT	0.28	2098
Fructose	-0.70	0.92	-0.10	-0.71	-1.67	1805
Fucopyranose	-5.24	-4.21	-2.17	-2.57	-2.45	1819
Galactofuranose	-0.09	-0.45	1.16	0.68	1.71	1913
Galactopyranose	-0.09	-0.11	1.30	-1.82	-0.82	1887
Galactose	-3.68	-0.43	-2.37	-3.24	-2.67	1998
Gluconic acid	6.24	4.32	6.97	5.72	5.50	1968
Glucose	2.37	0.18	3.36	2.05	3.06	1894
Glyceryl-glycoside	0.89	0.65	NDFT	2.00	1.08	2522
Guaiacol-ß-D-glucopyranoside	3.33	-0.34	0.26	3.43	2.91	2722
Gluconic acid-1,4-lactone	0.84	-0.48	1.15	NDFT	1.96	2195
Heptacosane	NDFT	2.54	NDFT	NDFT	2.32	2697
Hexadecanoic acid	NDFT	NDFT	NDFT	NDFT	NDFT	2592
Hexopyranose	-1.94	NDFT	-0.60	NDFT	0.87	2347
Hydromorphone	0.76	NDFT	0.54	NDFT	0.89	2346
Lactulose	4.03	3.49	1.79	3.72	5.29	2587
Maltose	-1.47	NDFT	1.27	-0.96	-5.01	2648
Mannose	NDZ	NDZ	NDZ	NDZ	NDZ	1813
Mannopyranose	-3.91	-0.52	-4.39	-3.66	-3.14	2012
Myo-Inositol	3.10	2.45	3.87	3.87	4.23	2110
N-Acetyl glucosamine	-1.21	-1.26	-1.66	-0.04	-1.75	2461
NI	NDZ	NDZ	NDZ	NDZ	NDZ	-
NI	2.50	1.36	3.28	3.21	1.96	-
NI	-3.41	NDFT	NDFT	NDFT	-0.96	-
NI	NDFT	0.66	NDFT	NDFT	-1.30	-
NI	-0.11	NDFT	-0.51	NDFT	1.41	-
Oleic acid	-3.62	NDFT	-0.68	NDFT	0.32	2233
Pentanedioic acid	NDFT	0.80	0.76	0.76	-0.39	1743
Psicofuranose	NDZ	NDZ	NDZ	NDZ	NDZ	1802
Ribonic acid	2.29	3.33	2.34	2.46	1.42	1764
Sorbitol	6.10	6.13	5.74	5.88	5.62	1924
Sorbofuranose	-0.73	-0.88	-1.78	-2.15	-3.79	1769
Stearic acid	1.61	-0.90	2.43	1.65	2.31	2782
Tetradecane	3.05	1.34	4.42	2.58	2.08	1706
Turanose	NDZ	NDZ	NDZ	NDZ	NDZ	2673
Tyrosine	NDZ	NDZ	NDZ	NDZ	NDZ	1928
Undecanedioic acid	NDZ	NDZ	NDZ	NDZ	NDZ	1853
Uridine	-0.67	NDFT	1.34	2.77	3.20	2411
Xylitol	4.49	1.67	5.56	5.88	6.16	1702
(E)-1-(3,5-Dimethoxyphenyl)-2-[4-(Octyloxy)phenyl]ethene	-0.38	-0.49	-0.01	1.66	1.13	2844
(S)-O-methylkreysigine	NDZ	NDZ	NDZ	NDZ	NDZ	2883
β-Gentiobiose	0.98	-2.35	-1.39	2.24	2.12	2853

^a^Fold change (FC) obtained by dividing the intensity of each metabolite from fermented samples (1 to 5 days) by that of unfermented samples and expressed as Log2 (FC).

NDZ = Metabolite not detected in unfermented samples. No Log2 (FC) was calculated.

NDFT = Metabolite not detected at the fermentation time. No Log2 (FC) was calculated.

NI = Metabolite not identified

The metabolites 2-butanone, and 2-undecanone were produced within the first 24 hours of fermentation. In addition, 2-pentanone appeared after 24 hours and its content increased until the end of fermentation. Other ketones were probably formed by catalyzed enzymatic reactions [49], and took part as precursors of secondary alcohols such as 2-heptanol and 2-pentanol [13]. In general, ketones showed slightly comparable abundances to aldehydes, but the content of ketones increased during the fermentation while aldehydes’ concentration decreased. Most of these compounds have been reported in fermented fine-flavor cacao beans [3, 13], fermented cacao clones [34], as well as cocoa liquor and chocolate prepared with Nacional cacao beans [5].

The esters which content significantly changed during fermentation (Fig 6) were represented by 2-octanol acetate, 2-pentanol acetate, and benzoic acid pentyl ester. The 3 compounds were detected in unfermented cacao beans and 2-pentanol acetate was the most abundant metabolite before to start fermentation. The content of esters notably increased after 24 hours of fermentation but decreased thereafter. In fact, benzoic acid pentyl ester was the only ester that remained detectable after 120 hours of fermentation (Fig 6). The esters formation during fermentation could be attributed to the esterification of their precursor alcohols such as the 2-pentanol [13, 50]. The 2-pentanol acetate has been reported in Criollo [3, 46], Nacional [3], and Trinitario [13] fermented cacao beans. However, 2-octanol acetate, and benzoic acid pentyl ester have been reported only for Criollo and Nacional varieties [3].

Regarding hydrocarbons which content significantly changed during fermentation (Fig 7), 2,4–diacetoxypentane was present in unfermented cacao beans, and their content appeared until the end of 48 hours of fermentation. Meanwhile, aromadendrene was found between 48–96 hours of fermentation, and trimethylacenaphthylene appeared after 72 hours, but its levels declined before completing the process. Similar patterns have been previously observed for aromadendrene during fermentation of Nacional and Criollo cacao beans [3].

Regarding other volatiles which content significantly changed during fermentation (Fig 8), 1,3-bis-t-butylperoxy-phthalan, 3-Formyl-N-methyl-9-[phenylethynyl]dibenzo[2,3-a:5,6-a](1,4)-thiazine, methane thiobis-, and n-hexylpentanamide were detected in unfermented cacao beans being methane thiobis- the most abundance compound. The 2-methyl-5H-dibenz[b,f]-azepine, 6-undecylamine, benzyl hydrazine, and caffeine were found at different intensities during the fermentative process, but their content declined before fulfilling the fermentation. Otherwise, the 3-trifluoroacetoxypentadecane was the only compound that remained after 5 days of fermentation. In particular, the 6-undecylamine has been shown a similar pattern during the fermentation of Nacional cacao beans [3].

Overall, most of the significant volatiles detected in this study provided desirable aromas, including 13 fruity, and 5 floral-like descriptors, which represents 50% of the total significant volatiles, and 32% of the total volatile compounds detected. However, odorless compounds such as caffeine as well as undesirable volatiles with camphoraceous (cyclohexanol), cheesy (heptanoic acid, pentanoic acid), fatty (hexanoic acid), and pungent (acetic acid, naphthalene, peracetic acid, propanoic acid) aromas were also found. Even so, additional sensory studies are required to know how each compound contributes globally to the aroma profile.

Various metabolites showed large errors bars, suggesting big biological variations between replicates. The spontaneous and uncontrolled nature of the cacao fermentation process likely contributed to the large variability observed in the data. Further research is needed to assess the concentration of metabolites using targeted approaches.

### Analysis of metabolites from liquid extracts

A total of 58 metabolites were detected in liquid extracts, of which 53 were putatively identified including 13 monosaccharides, 8 carboxylic acids, 5 fatty acids, 5 disaccharides, 3 amino acids, 3 polyols, 2 alkaloid derivatives, 2 hydrocarbons, 2 pyrimidines, 1 ketone, 1 alkaloid, 1 amine, 1 amino sugar, and 6 other compounds (Table 2). The data processing and compounds identification of the metabolites detected in liquid extracts of cacao samples at different fermentation times is detailed in S2 Table. This is the first report of metabolites from liquid extracts produced during on-farm fermentation of Nacional x Trinitario cacao beans.

**Fig 9:**
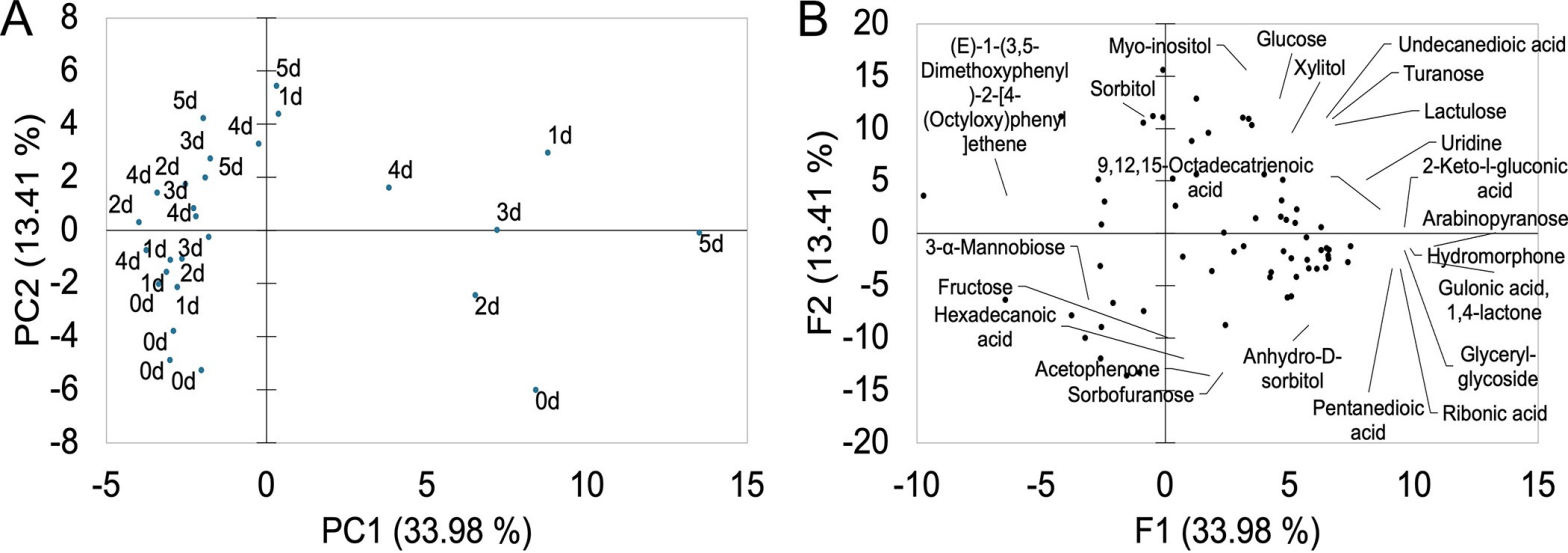
Principal components analysis (PCA) of metabolites in liquid extracts. Score plot (A) shows all samples and Loading plot (B) shows the metabolites with the highest loadings. Values in parenthesis represent the percentage contribution of PC1 and PC2 to the total variance.

**Fig 10:**
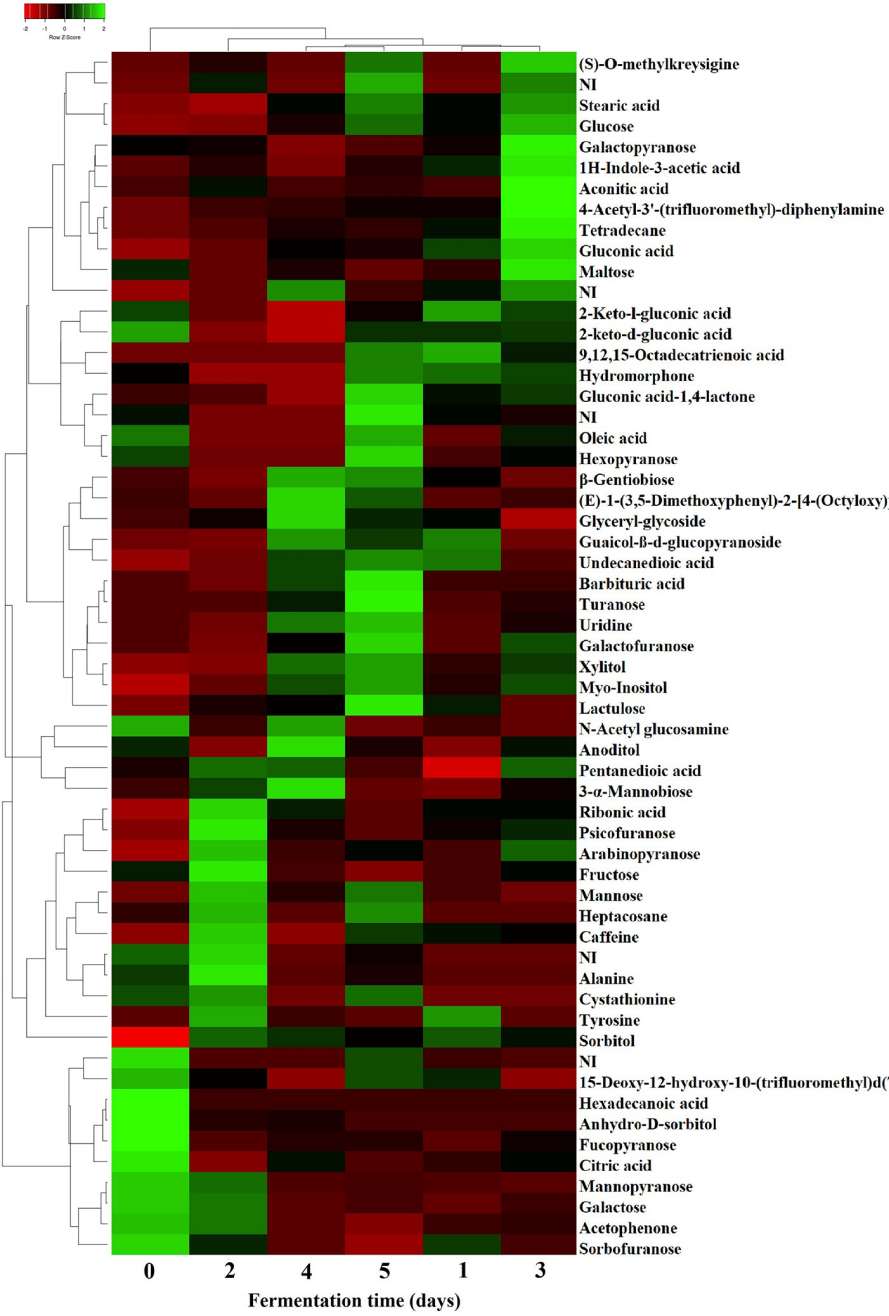
Heatmap analysis of metabolites from liquid-extracts during fermentation. The Heatmap illustrates the changes in the content of identified metabolites through 5-days of cacao bean fermentation.

Principal components analysis (PCA) of liquid extracts showed no grouping of samples according to the fermentation time (Fig 9) while HCA grouped samples from day 1 with day 3 and samples from day 4 with day 5 (Fig 10). Results suggest that metabolites from liquid extracts don’t fully explain the changes occurring during cacao fermentation. Fig 9B shows the loading plot of the metabolites with the highest contribution to the PCA. In general, unfermented cacao beans were characterized by the presence of 2-keto-D-gluconic acid, anhydro-D-sorbitol, acetophenone, citric acid, fucopyranose, galactose, N-acetyl glucosamine, hexadecenoic acid, and sorbofuranose. Then, the formation of 2-keto-L-gluconic acid, 9,12,15-octadecatrienoic acid, gluconic acid, guaicol-β-d-glucopyranoside, sorbitol, tyrosine, and undecanoic acid dominated the liquid extracts from samples taken during the first day of fermentation.

Meanwhile, 10 compounds including alanine, arabinopyranose, caffeine, cystathionine, fructose, heptacosane, mannopyranose, mannose, psicofuranose and ribonic acid showed high abundances at the second day of fermentation. Similarly, 1-H-Indole-3-acetic acid, (S)-O-methylkreysigine, 4-acetyl-3’-(trifluoromethyl)-diphenylamine, aconitic acid, galactopyranose, gluconic acid, glucose, maltose, stearic acid, and tetradecane were the most abundant compounds at the third day, as well as β-gentiobiose, (E)-1-(3,5-dimethoxyphenyl)-2-[4-(octyloxy)phenyl]ethene, glyceryl glycoside, guaiacol-ß-D-glucopyranoside, anoditol and 3-α-mannobiose at the fourth day. In contrast, the majority of compounds were detected at the fifth day including turanose, arabinopyranose, barbituric acid, galactofuranose, gluconic acid-1,4-lactone, hexopyranose, hydromorphone, lactulose, mannose, myo-inositol, oleic acid, uridine, undecanedioic acid and xylitol.

In general, sugars were the predominant metabolites in cacao liquid extracts during the complete fermentative process, including monosaccharides as glucose and fructose that probably were formed by hydrolysis of saccharides as effect of yeast fermentation in the pulp as well as by diffusion of yeast microbial activity products such as ethanol, acetic acid and lactic acid inside the cacao beans [11]. In fact, glucose and fructose contribute to the formation of aroma precursors after their reaction with peptides and amino acids [51] such as alanine and tyrosine, also detected in the present study. Additionally, the existence of peptides and reducing sugars (monosaccharides) contribute to the production of pyrroles, ketones and aldehydes during roasting via the Maillard reaction, improving the aroma quality of cacao beans and its derivatives [52]. Regarding fatty acids, the presence of unsaturated compounds such as oleic acid, possibly promotes the formation of lactones as degradation products due to reactivity of double-bounds [53], while saturated fatty acids such as palmitic acid, and stearic acid may be affecting the quality of cacao beans if found at higher levels [54], and could be responsible of rancid off-flavors [55]. However, oleic acid, palmitic acid, and stearic acid have been reported as the predominant fatty acids of fermented-dried fine-flavor cacao from Peru [56].

Acetophenone was detected in fresh and fermented samples suggesting that this molecule is an endogenous compound of the bean [13]. This amino acid-derived compound has been associated with floral and sweet aromatic notes and it has been reported in fermented-dried cacao beans of Criollo [11] and Trinitario [13] varieties as well as chocolate and cocoa liquor of Nacional cultivars [5]. Overall, the dynamic of GC-MS metabolites revealed the formation of most of the compounds after the second day of fermentation including four desirable compounds with floral (acetophenone), nutty (aconitic acid), sweet (maltose), and caramel (sorbitol) attributes representing the 6.8% of the total number of metabolites detected in the liquid extracts. Also, six odorless compounds including alanine, caffeine, citric acid, fructose, pentanedioic acid, and xylitol, as well as six undesirable metabolites with fatty (9,12,15-octadecatrienoic acid, oleic acid, stearic acid) and waxy (hexadecenoic acid, tetradecane) off-flavors were identified, each group accounting for the 10.3% of the total number of metabolites from the liquid extracts. The odor descriptors of the remaining metabolites were not found.

To the best our knowledge, the fermented Nacional x Trinitario cacao hybrid kept most of the volatile features corresponding to Nacional cacao beans. In particular, the floral attributes were enhanced with the presence of acetophenone and 2-pentanone, 5-phenyl-, which have not been reported for Nacional variety from the same origin [3]. Acetophenone has been described for dried-fermented Trinitario cacao beans [13] and derivatives of Nacional cacao hybrids [57] from different origins. These results confirmed that post-harvesting process have more influence on formation of aroma compounds than cacao variety and origin. However, further research focus on sensory profile is required to complement the odor descriptors of cacao beans during fermentation.

### Dynamics of individual metabolites from liquid extracts

**Fig 11:**
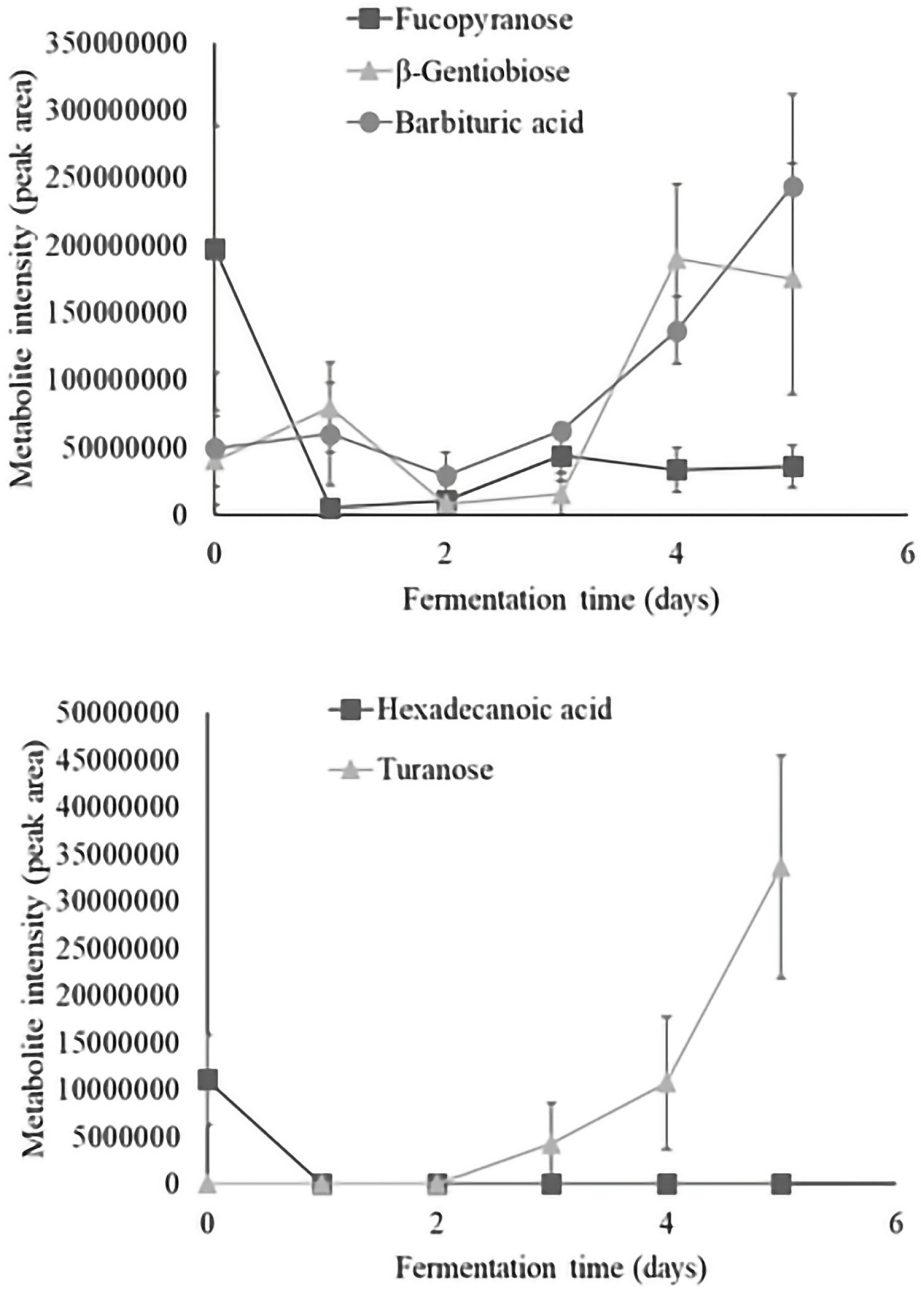
Dynamics of the metabolites from cacao liquid extracts which content significantly changed during fermentation (P<0.05). Levels of each significant metabolite from cacao liquid extracts are presented as metabolite intensity (peak area) through 5-days of cacao bean fermentation.

Time series analysis of individual metabolites revealed 5 significant compounds, including one monosaccharide, two disaccharides, one fatty acid, and one pyrimidine which dynamics are illustrated in Fig 11 and described below.

Regarding monosaccharides which content significantly changed during fermentation, the fucopyranose appeared in fresh cacao beans, and showed abundance reduction after fermentative process, which was mainly evident at the beginning of fermentation. This phenomenon could be explained by the yeast consumption of sugars from the pulp that mainly occurs during first days of fermentation [11, 42]. In fact, the metabolite intensity patterns of the fucopyranose, as well as anhydro-D-sorbitol, and sorbofuranose illustrated in Fig 11 showed declines between 0–24 hours of fermentation. Regarding disaccharides which content significantly changed during fermentation, the turanose was formed within the first 24 hours of fermentation and its contents incremented after completing the fermentation period. Concerning the significant fatty acids, hexadecanoic acid was identified in unfermented cacao beans but the metabolite intensity rapidly declined within the first 24 hours.

Despite not being a compound with significant changes throughout the fermentation, the citric acid was the most abundant metabolite detected in the liquid extracts of unfermented cacao beans. This carboxylic acid has been reported to represent around 1% of the cacao pulp composition [46]. Then, the reduction of citric acid afterwards was probably associated with the ability of yeasts to metabolize citric acid during fermentation to produce esters [34]. Also, citric acid can be converted to lactic acid, ethanol, formic acid, acetic acid, and some flavor precursors such as ketones and aldehydes after metabolization by lactic acid bacteria [58].

### Analysis of antioxidant capacity and anthocyanin content

**Fig 12:**
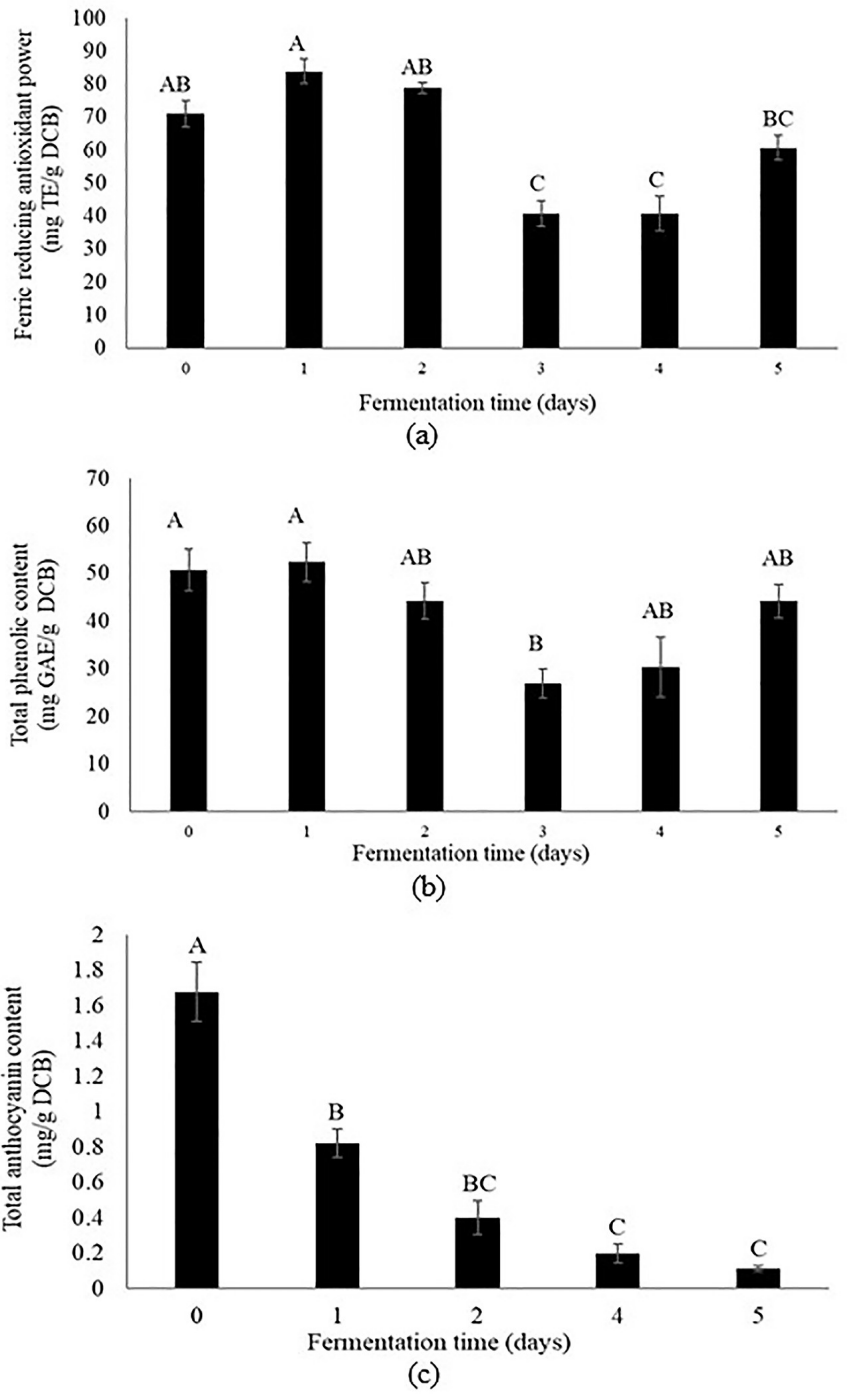
Ferric reducing antioxidant power (a), total phenolic content (b), and anthocyanin content (c) during fermentation. Values are expressed as mean ± standard deviation (n = 3). The same letter indicates no significant differences among fermentation days (Tukey test, *p*<0.05).

The total phenolic content (TPC), the ferric reducing antioxidant power (FRAP), and the anthocyanins content of on-farm cacao beans during fermentation are presented in Fig 12. The anthocyanin content varied from 1.68 (unfermented) to 0.11 (day 5) mg CGE/g DCB, while the antioxidant capacity ranged from 83.86 (day 1) to 40.63 (day 3) mg CGE/g DCB. The similar pattern such as FRAP assay was observed for TPC values, and the highest value was found in day 1 (52.42 mg GAE/g DCB), whereas the lowest was observed in day 3 (26.92 mg GAE/g DCB).

The total anthocyanins content declined during fermentation process, and the highest values were shown by unfermented cacao beans. These results agree with previous reports showing that cyanidin 3-galactoside levels in Trinitario cacao beans from Cuba and Cameroon decrease after 5 days of fermentation [18]. In the same way, the total anthocyanin content of Colección Castro Naranjal 51 (CCN-51) cacao beans cultivated in Peru decreased from 13.26 to 1.78 mg cyanidin-3-glucoside/g after 7 days of fermentation [59]. Similarly, this pattern has been reported for fermented-dried cacao beans of Nacional (Ecuador), and Trinitario (Venezuela) varieties. In this case, cyanidin 3-*O*-galactoside content showed reductions of 0.26 and 0.32 mg/g respectively [17]. In fact, the cyanidin 3-galactoside content of the Ecuadorian variety decline from 0.117 to 0.003 mg/g in the process from fermented cacao beans to dark chocolate. Interestingly, the samples from Ecuador showed the highest levels of this metabolite in comparison with those from Nigeria, Cameroon, Ivory Coast, and Ghana [60]. As expected, the total anthocyanins content of fermented cacao beans decreased during fermentation since the content of anthocyanins as well as color hue have been used as indicators of the level of fermentation. Thus, the reduction observed in this study could be attributed to pigments conversion into cyanidins and sugars due to hydrolysis reactions during fermentation [61]. Regarding to TPC, results were similar to those described for fermented-dried Trinitario cacao beans from Nicaragua (43 mg GAE/g) [15], lower than data reported for Trinitario cacao beans from Costa Rica (100–120 mg GAE/g) [16], and Brazilian Forastero variety (55.00–89.80 mg GAE/g) [62]. Additionally, these findings show that reducing capacity of phenolics decline when comparing the complete fermented samples (44.20 mg GAE/g DCB) with the unfermented beans (52.42 mg GAE/g DCB), and similar results have been informed for CCN-51 cacao beans, as well as in the process from fermented cacao beans to dark chocolate (44.7 to 14.9 mg catechin equivalents/g) [60]. In particular, the TPC revealed a positive linear correlation with the results of the FRAP assay (r  =  0.908, *p* < 0.05), suggesting that FRAP values could be explained partially by the presence of phenolic compounds responsible of reducing capacity. Other studies have corroborated the existence of such correlation in fermented cacao beans [16, 62], and chocolate prepared with different cacao content [63]. The FRAP values of the present study are higher than information described for dark chocolate made with blend cacao mass originating from different Latin American countries (37.96 g Trolox equivalents per kg) [63].

Notably, the tendency of FRAP values obtained in this work is comparable with data reported for Trinitario cacao beans of Costa Rica in which the ferric reducing antioxidant capacity values fluctuated around 700–900 μmol FE^2+^/g during 5 days of fermentation with trend to increase at the end of the fermentation [16]. Other studies of Brazilian Forastero variety have been reported higher values for fermented-dried cacao beans (1483 μmol Fe^2+^/g) [62], as well as lower for chocolate made with fermented-dried-roasted cacao beans (530.06 μmol FE^2+^/g) [64], denoting the effect of post-harvest practices on cacao beans. In the present study, the FRAP values of 5-day fermented Nacional x Trinitario cacao beans were lower than unfermented cacao beans. As indicated before, the ferric reducing antioxidant capacity is significantly correlated to total phenolic content, and similar trends were found in both assays. In this context, probably the total phenolic content could be explained by the transformation of polyphenols to insoluble tannins due to enzymatic degradation and heat conditions of fermentation and drying processes [15, 54, 65], as well as polyphenols diffusion outside the beans due to cell damage that arises during fermentation [66].

In summary, the assessed post-harvesting process affected the bioactivity of antioxidant compounds after 5 days of fermentation. As mentioned before, this similar trend has been observed independently of cacao variety and origin [14–16]. So, this study highlights the effect of spontaneous fermentation on phenolic acids and anthocyanins of Nacional x Trinitario cacao beans.

## Conclusions

This study revealed that dynamics of the main aroma compounds of Nacional x Trinitario cacao beans was defined by the formation of various metabolites after 48 hours of fermentation. The identified desirables compounds include 17 fruity, and 9 floral-like volatiles as well as metabolites with caramel, chocolate, ethereal, nutty, sweet, and woody aromatic notes. Nevertheless, undesirable metabolites with camphoraceous, cheesy, fatty, and pungent attributes were also found. The detected compounds that were formed or degraded during the processes were possibly derived from the pulp, endogenous of the bean or synthesized by microorganisms. Furthermore, the anthocyanins content showed a reduction over time, while FRAP-TPC values exposed variations during fermentation and notably similar tendency denoting a partial correlation between the assays. These findings contribute to the understanding of the aroma compounds development and the antioxidant activity during the fermentative process, which could be used in future works in order to improve the fermentation and subsequently the quality of fermented cacao beans.

## Supporting information

S1 Table Metabolites detected in the headspace of cacao samples. Data processing, compounds identification and aroma descriptors of the metabolites detected in the headspace of cacao samples at different fermentation times.
(XLSX)

S2 Table Metabolites detected in the liquid extract of cacao samples. Data processing and compounds identification of the metabolites detected in liquid extracts of cacao samples at different fermentation times.
(XLSX)
